# Relationships between sensory stimuli and autonomic nervous regulation during real and virtual exercises

**DOI:** 10.1186/1743-0003-4-38

**Published:** 2007-10-06

**Authors:** Tohru Kiryu, Atsuhiko Iijima, Takehiko Bando

**Affiliations:** 1 Graduate School of Science and Technology, Niigata University, 8050 Ikarashi-2, Nishi-Ku, Niigata 950-2181, Japan; 2 Center for Transdisciplinary Research, Niigata University, 8050 Ikarashi-2, Nishi-Ku, Niigata 950-2181, Japan; 3 Graduate School of Medical and Dental Sciences, Niigata University, 1-757 Asahimachi-dori, Chuo-Ku, Niigata 951-8520, Japan

## Abstract

**Background:**

Application of virtual environment (VE) technology to motor rehabilitation increases the number of possible rehabilitation tasks and/or exercises. However, enhancing a specific sensory stimulus sometimes causes unpleasant sensations or fatigue, which would in turn decrease motivation for continuous rehabilitation. To select appropriate tasks and/or exercises for individuals, evaluation of physical activity during recovery is necessary, particularly the changes in the relationship between autonomic nervous activity (ANA) and sensory stimuli.

**Methods:**

We estimated the ANA from the R-R interval time series of electrocardiogram and incoming sensory stimuli that would activate the ANA. For experiments in real exercise, we measured vehicle data and electromyogram signals during cycling exercise. For experiments in virtual exercise, we measured eye movement in relation to image motion vectors while the subject was viewing a mountain-bike video image from a first-person viewpoint.

**Results:**

For the real cycling exercise, the results were categorized into four groups by evaluating muscle fatigue in relation to the ANA. They suggested that fatigue should be evaluated on the basis of not only muscle activity but also autonomic nervous regulation after exercise. For the virtual exercise, the ANA-related conditions revealed a remarkable time distribution of trigger points that would change eye movement and evoke unpleasant sensations.

**Conclusion:**

For expanding the options of motor rehabilitation using VE technology, approaches need to be developed for simultaneously monitoring and separately evaluating the activation of autonomic nervous regulation in relation to neuromuscular and sensory systems with different time scales.

## Introduction

It takes a long time for functional recovery in motor rehabilitation, and providing appropriate tasks and/or exercises during the progression of recovery is necessary to continue promoting motor rehabilitation with sufficient effectiveness, as well to motivate the patient. Current virtual reality (VR) and virtual environment (VE) technologies are now being applied to rehabilitation engineering [1] because they are expected to help restore the sensory and physical functions without any restriction in the real world. Application of a VE to motor rehabilitation expands the number of options for selecting rehabilitation tasks and/or exercises including real active exercise, real passive exercise, active or passive exercise in a VE, electrical or mechanical stimulation for paralyzed muscles, and visual stimulation with a first-person-view video image.

However, enhancing or augmenting a specific sensory stimulus in a VE sometimes causes unpleasant sensations due to conflicts among sensory stimuli (sensory conflict theory [2]). This problem in a VE has been referred to as "cybersickness" in relation to simulator sickness and motion sickness [3,4]. That is, unbalanced stimuli that are different from those experienced in the real world sometimes cause unpleasant sensations, even though they are expected to increase the feeling of reality. Studies have described unpleasant sensations in the application of VR and VE technologies in motor rehabilitation [5,6], and researchers have studied unpleasant sensations using subjective indices [7] and autonomic-nervous-activity-related indices [8]. Repetitive muscle activity in the real exercise produces physical fatigue like unpleasant sensations in the virtual task, and physical fatigue could be evaluated by using autonomic nervous regulation.

Physical activity mainly consists of several functional components with different time scales. Autonomic nervous activity (ANA) dominantly regulates the person's physical conditions after exercise or exercise-related sensory stimuli for several seconds. In contrast, muscle activity and sensory activity work within a few tens of milliseconds. When selecting appropriate tasks and/or exercises for individuals, we should consider the relationship between ANA and sensory stimuli.

We conducted a feasibility study of using autonomic nervous regulation in response to several sensory stimuli, for real cycling and for virtual mountain biking using a first-person-view video image. We estimated the ANA from the R-R interval time series of electrocardiogram (ECG) and incoming sensory stimuli that would activate the ANA. To evaluate the exercise-related factors in real exercise, we measured vehicle data and electromyogram (EMG) signals during the cycling exercise. We measured the eye movement of the subject in relation to image motion vectors while he or she was viewing the first-person-view vection-inducing mountain-bike video images. Although muscle contractions generally elicit a strong demand on the ANA, visual stimuli are not always the strong demand for everyone. Accordingly, we carefully considered where and when the incoming stimuli and the ANA should be evaluated.

## Methods

**Figure 1:**
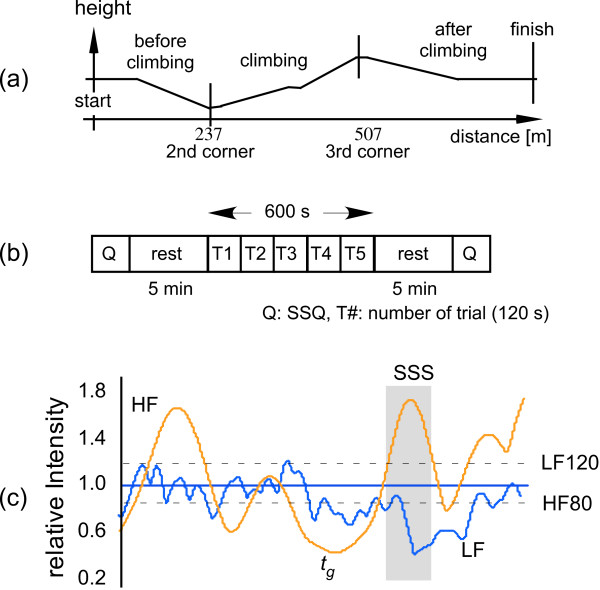
Evaluation process of autonomic regulation for incoming stimuli: (a) overview of circuit path for real exercise; (b) sequence of trials for virtual exercise; (c) definition of *t*_*g *_and SSS for virtual exercise.

Since autonomic nervous regulation should be evaluated after incoming stimuli, we focused on the specific sections before and after climbing a hill on a bicycle in the real world (Figure 1(a)) and the sections specified by the behavior of ANA-related indices in the virtual world (Figure 1(c)).

### Experimental procedure

The subjects were volunteers and were informed of the risks involved and signed a consent form in advance, and were free to withdraw at any time during the experiment. For biosignal processing, a time interval of over a few minutes is necessary to estimate the ANA, even though the exercises or exercise-related sensory stimuli are very short events. A trial consisted of a series of events followed by enough rest to estimate the ANA.

For the real exercise [9], the subjects were asked to pedal a torque-assisted bicycle at 60 rpm for as long as possible. The length of the path was approximately 840 meters, with a steep uphill section near the middle; the maximum incline was 5.7 deg (Figure 1(a)). We divided the path into three phases: before and after climbing, and climbing. An experimental set consisted of six consecutive trials, and each trial comprised a 2.5-min cycling exercise followed by a 2-min rest. The ECG and EMG signals were measured using a tablet PC and were sampled at 5000 Hz with 12-bit resolution. We also measured the speed, cadence, and torque as vehicle data and compared them with the muscle activity for every pedal stroke.

For the virtual exercise [10,11], the subjects continuously viewed a 2-min-long mountain-bike video taken from a first-person viewpoint, five times for 10 min, followed by a 5-min rest (Figure 1(b)); the video camera had been mounted on the handlebars of a mountain bike, and it sometimes produced off-centered vection or random camera shake. The video image was back-projected onto an 80-inch screen by XGA video projectors with over 2500 ANSI lumens, and the illumination in the room was 10 lx. The distance between the subject and screen was about 2 meters, resulting in horizontal and vertical view angles of 22 and 17 deg, respectively. We recorded the ECG, and measured the blood pressure using the tonometry method, the respiration using strain sensors around the chest and abdomen for use as ANA-related biosignals, and the eye movement for evaluating sensory activity by using a limbus tracker, at a sampling frequency of 1000 Hz with 12-bit resolution.

### Biosignal processing

At the sensory systems level, we used the correlation coefficient to derive the relationship between the external sensory stimuli and those responses due to different time scales. For evaluating the response to external sensory stimuli, we compared the behavior of ANA-related indices before and after the stimuli.

In the real exercise, the strongest demand on ANA was from muscle contractions. We used the average rectified value (ARV) from the EMG signals as a muscle-force-related index [9,12], then calculated the correlation coefficient between ARV and pedal torque, γ_ARV-trq_. As a muscle fatigue-related index, we used the mean power frequency (MPF) from the EMG signals and calculated the correlation coefficient between ARV and MPF, γ_ARV-MPF_. These correlation coefficients were obtained from samples estimated with a sliding 50-ms interval every 25 ms during each pedal-stroke interval of 400 ms. Surveying the results from around 200 contractions for each trial, we selected five consecutive pedal strokes immediately before the hilltop during climbing and averaged γ_ARV-trq _and γ_ARV-MPF_. Grouping was done for every trial with γ_ARV-MPF _and ANA-related indices. We estimated the ANA-related indices for each phase from the R-R interval time series by using the continuous wavelet transform. For the real exercise, the focused frequency band was related to the respiratory sinus arrhythmia (RSA) [13], which had a frequency band ranging from 0.3 to 0.6 Hz during exercise. In practice, we calculated the power ratio of RSA, (total power at 0.3–0.6 Hz)/(total power at 0.01–1.25 Hz), and then averaged it for each phase to discriminate trials in relation to the autonomic nervous regulation property. We denote the averaged power ratio of RSA as *pr*_RSA_.

For the virtual exercise, several types of biosignals were available during the experiments, which were done in the laboratory. To quantify the input visual stimuli, we estimated the zoom, pan, and tilt components of the global motion vector (GMV) of the video images: GMV is a key technology in image data compression [14]. We calculated the correlation coefficient between the GMV and eye movement, γ_GMV-eye_, every 10 s as the sensory response. Identifying the input visual stimuli for unpleasant sensations is difficult because they are relatively weak. We obtained a time-varying ANA-related indices for every frame (30 frame/s) with a 10-s interval from the R-R interval, the respiration, and the blood pressure time series by using the continuous wavelet transform. The focused frequency bands of the indices were 0.04–0.15 Hz (Mayer wave related low-frequency (LF) band) and 0.16–0.45 Hz (RSA related high-frequency (HF) band). The LF and HF components for five consecutive tasks were further normalized using the average LF and HF components estimated during a preceding rest period, respectively. We then defined some sensation section (SSS) on the basis of three ANA-related conditions [10]: the LF component is greater than 120% of the average LF component, the HF component is less than 80% of the average HF component, and the length of the SSS is over 300 msec (Figure 1(c)). Next, we determined the trigger point of the SSS, *t*_*g*_, by searching the local minimum of the LF component backwards in time. To screen the visually induced sensation before and after the video viewing (Figure 1(b)), we used the total severity scores of the Simulator Sickness Questionnaire (SSQ) [7]. The total SSQ score is a combination of components based on the levels of nausea, oculomotor problems, and disorientation.

## Results

### Real exercise

The participants in the real exercise were 13 healthy volunteers (eight men and five women, 20.0 ± 0.8 years). Using *pr*_RSA _and γ_ARV-MPF_, we classified the 103 trials into four groups. First, we set the threshold at 20% of the average *pr*_RSA _before climbing, estimating the median (21.3%) from all the trials. For a high-percentage *pr*_RSA _(HRSA) before climbing, a large fluctuation occurred in the R-R interval before and after climbing, specifically during the rest periods [9]. A little fluctuation occurred in the R-R interval before and after climbing for a low-percentage *pr*_RSA _(LRSA) before climbing. Second, we used the γ_ARV-MPF _immediately before the hilltop because the samples for the positive γ_ARV-MPF _region showed the largest shift in *pr*_RSA _in relation to climbing efforts. During the first half of a peal stroke, positive and negative γ_ARV-MPF _means increasing muscle activity and muscle fatigue, respectively [9,12]. During the second half, positive and negative γ_ARV-MPF _means decreasing and disappearing muscle activity, respectively. We represent positive γ_ARV-MPF _as increasing or decreasing muscle activity (I/D) and negative γ_ARV-MPF _as fatigue or disappearing muscle activity (F/D).

**Figure 2:**
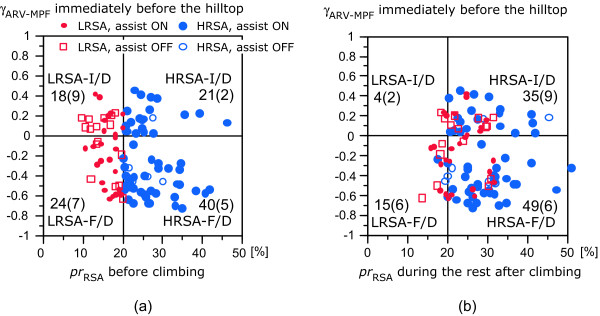
Scatter graphs between *pr*_RSA _and **γ**_**ARV-MPF **_during climbing for four categories: (a) *pr*_RSA _before climbing; (b) *pr*_RSA _during the rest after climbing. The number of samples for each group is displayed with the number of power-assist-off trials in parentheses.

We categorized each trial into one of four groups on the basis of the median of *pr*_RSA _and the sign of γ_ARV-MPF _for five consecutive pedal strokes immediately before the hilltop and plotted the results in a scatter graph (Figure 2). Table 1 presents the results for other indices. The four groups are denoted HRSA-I/D, HRSA-F/D, LRSA-I/D, and LRSA-F/D. In Figure 2(a), the percentage of power-assist-off trials was the highest for LRSA-I/D and the lowest for HRSA-I/D. The results for HRSA-F/D, which had the largest number of trials, showed negative γ_ARV-MPF _with a high *pr*_RSA_; even in HRSA with power-assist-on trials, negative γ_ARV-MPF _sometimes occurred. In this group, the speed was medium, and the torque was the lowest (Table 1). In contrast, the results for LRSA-F/D showed negative γ_ARV-MPF _and LRSA. The speed was the lowest, and the torque was medium. In the LRSA-I/D group, the speed was close to that of the HRSA-F/D group and the torque was larger than those of the HRSA-F/D and the LRSA-F/D groups. The highest speed and torque with positive γ_ARV-MPF _was for HRSA-I/D. As shown in Figure 2(b), the *pr*_RSA _during the rest after climbing was significantly higher than *pr*_RSA _before climbing (paired *t*-test, *p *< 0.05), especially for the HRSA-I/D group. Contrary to our expectation for torque-assisted bicycles, the torque-assist supported the appearance of HRSA, but it was sometimes not enough for muscle fatigue.

**Table 1:** Speed, torque, γ_ARV-MPF_, and γ_ARV-trq _during climbing for four groups.

	HRSA-I/D	HRSA-F/D	LRSA-I/D	LRSA-F/D
speed [km/h]	18.3 ± 2.1	16.4 ± 2.4	16.2 ± 2.5	15.9 ± 2.2
torque [Nm]	31.0 ± 8.6	23.4 ± 7.1	29.2 ± 7.9	25.3 ± 6.1
γ_ARV-MPF_	0.19 ± 0.33	-0.47 ± 0.15	0.18 ± 0.11	-0.34 ± 0.21
γ_ARV-trq_	0.66 ± 0.18	0.62 ± 0.33	0.66 ± 0.21	0.64 ± 0.22

### Virtual exercise

**Figure 3:**
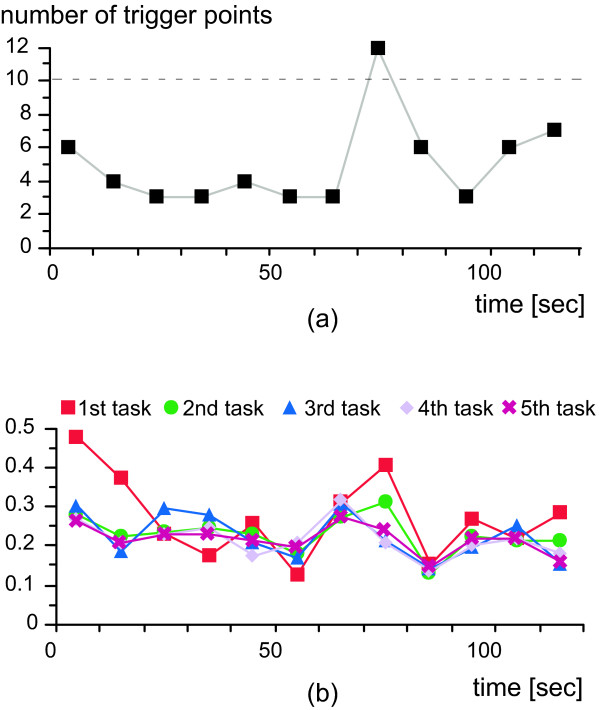
Time distributions of trigger points and **γ**_**GMV-eye **_for each 10-s segment for 2-min-long randomly camera-shaken video image: (a) number of trigger points accumulated for five tasks; (b) γ_GMV-eye _for each task averaged among "unpleasant" group. Note that the pan component and the horizontal movement were used as the GMV and the eye movement, respectively.

**Figure 4:**
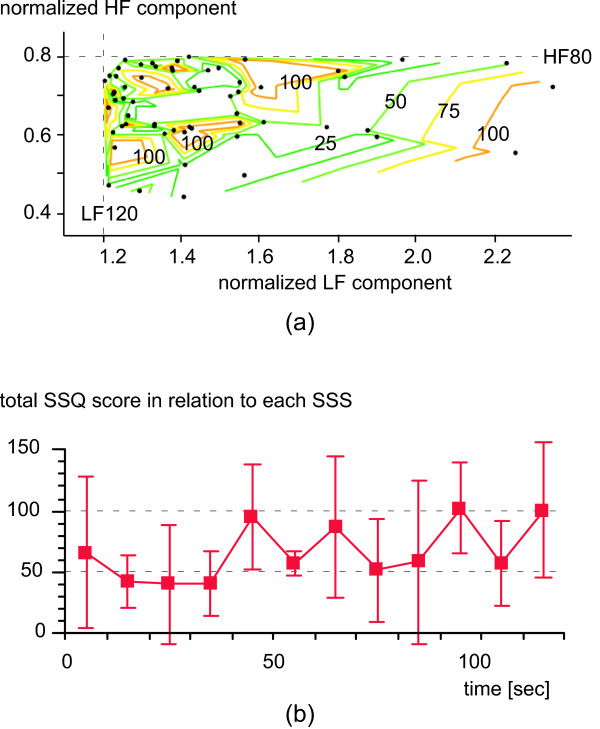
Distributions of total SSQ score in relation to SSS: (a) contour plot of total SSQ score as a function of normalized LF and HF components at each SSS (60 dots); (b) practical time distribution of the total SSQ score in relation to each SSS.

Fifteen healthy men (21.9 ± 0.9 years) voluntarily participated in the virtual exercise. Nine experienced unpleasant sensations while watching the mountain-bike video and six did not, as classified by their total SSQ scores. The time distribution of the total 60 trigger points for each 10-s segment is shown in Figure 3(a). The trigger points were concentrated in the 71–80-s segment of the 2-min-long video image. As shown in Figure 3(b), γ_GMV-eye _showed the similar behavior to the trigger points for the first task, but did not after the second task. That is, around the 71–80-s segment, the subjects' eyes relatively followed the camera motion for the first task (γ_GMV-eye _= 0.4), but γ_GMV-eye _decreased after the second task. This did not occur for the "non-unpleasant" group. Figure 4(a) shows a contour plot of the total SSQ score as a function of the normalized LF and HF components for all the 60 samples at each SSS. The total SSQ score was higher than 100 in a few regions far from the thresholds of the ANA-related conditions. That is, the ANA-related conditions determining the SSS covered the subjects with a high total SSQ score. Moreover, the total SSQ score in relation to each SSS practically revealed the time distribution of the total SSQ score (Figure 4(b)): for each 10-s segment, we estimated the mean and standard deviation of the total SSQ score among related subjects in relation to each SSS. The total SSQ score for the 61–120-s segment was significantly higher than that for the 1–60-s segment (*t*-test, *p *< 0.05).

## Discussion

A virtual environment increases the number of options for selecting approaches to motor rehabilitation. The options range from active exercise in the real world with muscle contractions to passive exercise in the virtual world with visual stimulation. To provide appropriate tasks and/or exercises for individuals undergoing motor rehabilitation, we focused on the impact of external sensory stimuli and autonomic nervous regulation as their responses.

Autonomic regulation is important in a series of repetitive exercises because it controls the cardiovascular system. During real exercise, the muscle sympathetic nerve activity activated by strong muscle contractions elicits autonomic nervous responses [15,16]. This autonomic regulation supports continuous real exercise. A low γ_ARV-trq _reflects a mismatch between muscle activity and pedal torque resulting from poor pedaling skills. This mismatch could increase muscular fatigue, resulting in strong requests for autonomic regulation after climbing, even with the power-assist on. However, γ_ARV-trq _did not significantly differ among the four groups (Table 1), so the difference in *pr*_RSA _was not fully linked to muscular fatigue (Figure 2). Refer to [9] for supplemental results. Thus, evaluation of muscle activity separately from *pr*_RSA _is necessary for preventing muscular fatigue in motor rehabilitation.

In contrast, the ANA was more difficult to distinguish during virtual exercise than during real exercise, according to the results for the first-person-view vection-inducing video images. We defined the ANA-related conditions and obtained a remarkable time distribution of trigger points that would evoke unpleasant sensations in ANA (Figure 3(a)). Around the trigger points, we have obtained the related time-frequency components of the GMVs ranging from 0.3 to 2.5 Hz [11]. The correlation coefficient between GMV and eye movement around the peaks of trigger points was 0.4 for the first task and decreased after the second task for the unpleasant group (Figure 3(b)). This might have been caused by the progression of the mismatch between the specific time-frequency structure of the GMVs and eye movement. Thus, evaluation by sensory features could be useful for specifying sensations in addition to ANA-related indices. The SSS derived from the ANA-related conditions enabled us to evaluate the distribution of the total SSQ score as a function of the LF and HF components (Figure 4(a)). Moreover, the SSS eventually represented the time distribution of the averaged total SSQ score (Figure 4(b)). Since the SSQ reflects the oculomotor problems and disorientation as well as the levels of nausea, Figure 4(b) obtained by the ANA-related conditions did not fully explain the behavior of sensations. However, those approaches have a potential in revealing the event-related autonomic response for a weak stimulus like a visual one. We will compare the total SSQ score with the sensory activity as a function of time in the next step.

The level in the disturbance of autonomic regulation depends on the individual. Therefore, to provide appropriate tasks and/or exercises as recovery progresses, we need to simultaneously monitor and separately evaluate the neuromuscular and sensory systems and autonomic regulation as their responses. Appropriate tasks and/or exercises in motor rehabilitation will properly activate the ANA by neuromuscular and sensory systems: muscle contractions in real exercise and visual stimuli in virtual exercise are trigger factors. The autonomic nervous system receives many types of stimuli from different sensory systems with different time scales and seems to set individual priority for autonomic responses. Since the threshold between positive and negative effects would vary even for the same stimuli, depending on the behavior of autonomic nervous regulation, the differences between real and virtual exercises should be studied in terms of the ANA-related indices. We did a preliminary cross-validation study between real and virtual exercises for the same nine subjects, but we have not yet identified specific features. Further cross-validation studies should provide hints for designing continuous repetitive training or exercises for motor rehabilitation.

## Conclusion

We investigated the process of repetitive training or exercises to be used for continuous motor rehabilitation with sufficient effectiveness and motivation by comparing real and virtual exercises. The evaluated factors were muscle activity and vision properties depending on the type of task and exercises as well as the autonomic nervous activity estimated from the heart rate variability. Our results showed that fatigue in the real world should be evaluated on the basis of not only muscle activity but also autonomic nervous regulation after exercise. Moreover, unpleasant sensations in the virtual world should be checked first in terms of vision properties and then in terms of autonomic nervous regulation. To expand the options for motor rehabilitation using virtual environment technology, we need to develop approaches for simultaneously monitoring and separately evaluating the activation of autonomic nervous regulation in relation to neuromuscular and sensory systems with different time scales.
